# Complete response in a patient with lung cancer suffering from three pembrolizumab‐induced immune‐related adverse events including retinal vasculitis

**DOI:** 10.1002/rcr2.730

**Published:** 2021-03-07

**Authors:** Chihiro Mimura, Motoko Tachihara, Sentaro Kusuhara, Hidenori Fukuoka, Yoshihiro Nishimura

**Affiliations:** ^1^ Division of Respiratory Medicine, Department of Internal Medicine Kobe University Graduate School of Medicine Kobe Japan; ^2^ Division of Ophthalmology, Department of Surgery Kobe University Graduate School of Medicine Kobe Japan; ^3^ Division of Diabetes and Endocrinology, Department of Internal Medicine Kobe University Graduate School of Medicine Kobe Japan

**Keywords:** Hypophysitis, immune‐related adverse events, lung cancer, retinal vasculitis, thyroiditis

## Abstract

A 71‐year‐old man was diagnosed with squamous cell lung carcinoma with high expression of programmed cell death ligand 1 (PDL1) (cT4N1M1b stage IVA). He was treated with pembrolizumab, but 14 days later, he suffered from pembrolizumab‐related retinal vasculitis as an immune‐related adverse event (irAE). The symptoms were ameliorated by oral corticosteroids. We succeeded in switching to topical treatment as early as possible with the help of an ophthalmologist. Six months after discontinuing treatment with oral prednisolone, hypophysitis and thyroiditis occurred in six cycles of pembrolizumab. Finally, he suffered from three irAEs, but the antitumour effect resulted in a remarkable response.

## Graphical abstract

We report a case of non‐small cell lung cancer with a long‐term response associated with multiple immune‐related adverse events caused by pembrolizumab, such as retinal vasculitis, hypophysitis, and thyroiditis.

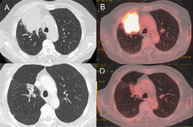

## Introduction

In recent years, immune checkpoint inhibitors (ICIs) have played an important role in the treatment of lung cancer. However, immune‐related adverse events (irAEs) caused by ICIs are known to cause various medical conditions, and some of them may become serious. It is difficult to predict the time of onset and rare diseases may occur. We report a case of non‐small cell lung cancer (NSCLC) with a long‐term response associated with multiple irAEs caused by pembrolizumab, such as retinal vasculitis, hypophysitis, and thyroiditis.

## Case Report

**Figure 1:**
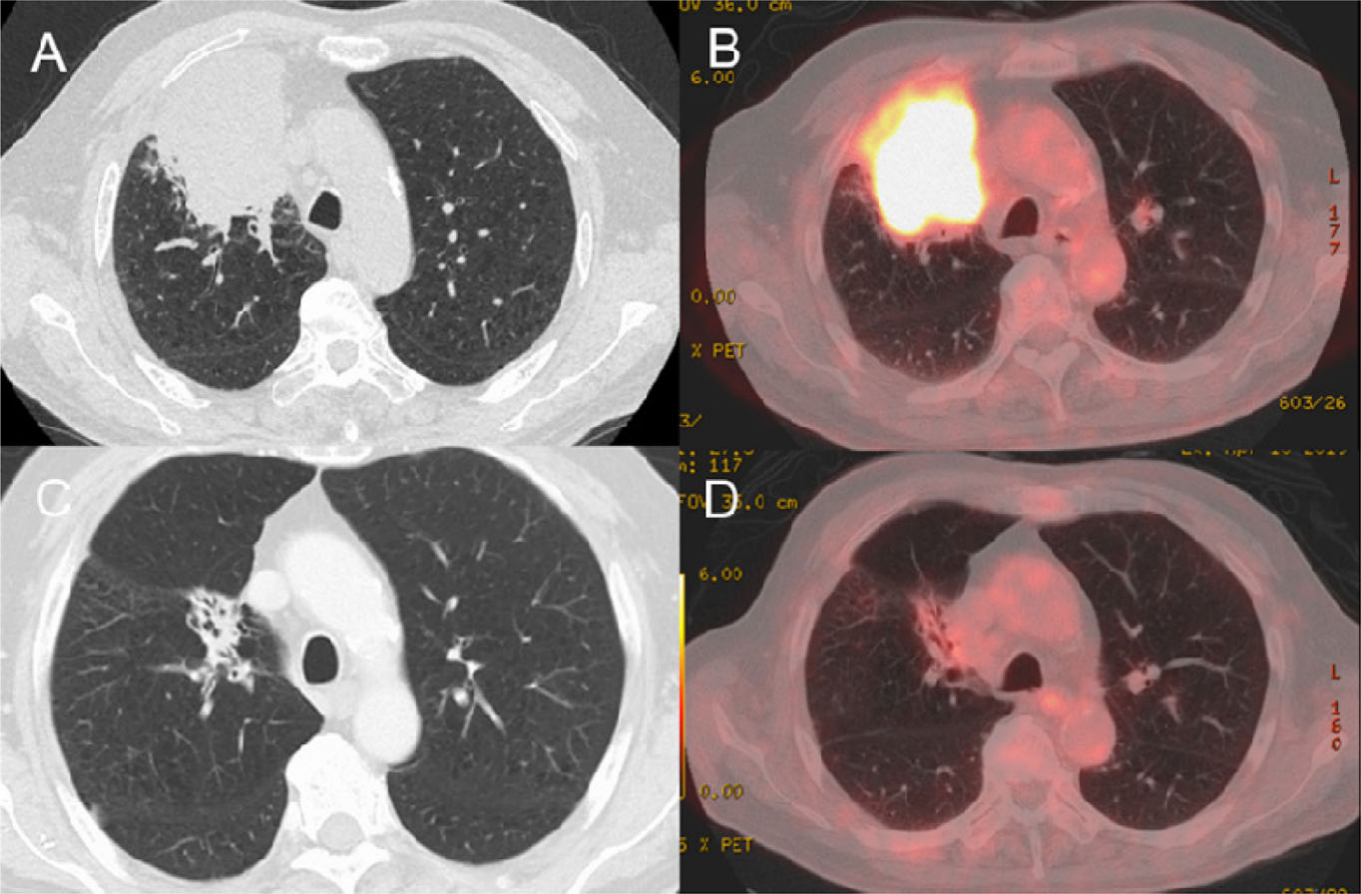
A mass with a size of 85 mm is shown on chest computed tomography (CT) (A). Intense uptake is shown in the lesion visualized with 18F‐fluorodeoxyglucose positron emission tomography (PET) (B). Chest CT and PET‐CT show a significant reduction in the size of the mass compared with that observed 15 months earlier (C,D).

A 71‐year‐old man with a 76.5 pack‐year smoking history presented with a four‐month history of cough. He was referred to our hospital to examine abnormal shadows on chest X‐ray in the upper right lobe. He was clinically diagnosed with squamous cell carcinoma with high expression of PD‐L1 by bronchoscopy. A routine metastatic work‐up revealed his clinical stage to be IVA (cT4N1M1b, adrenal metastasis) (Fig. 1A, B). He was treated with 200 mg pembrolizumab per dose, but 14 days later, he experienced blurred vision.

**Figure 2:**
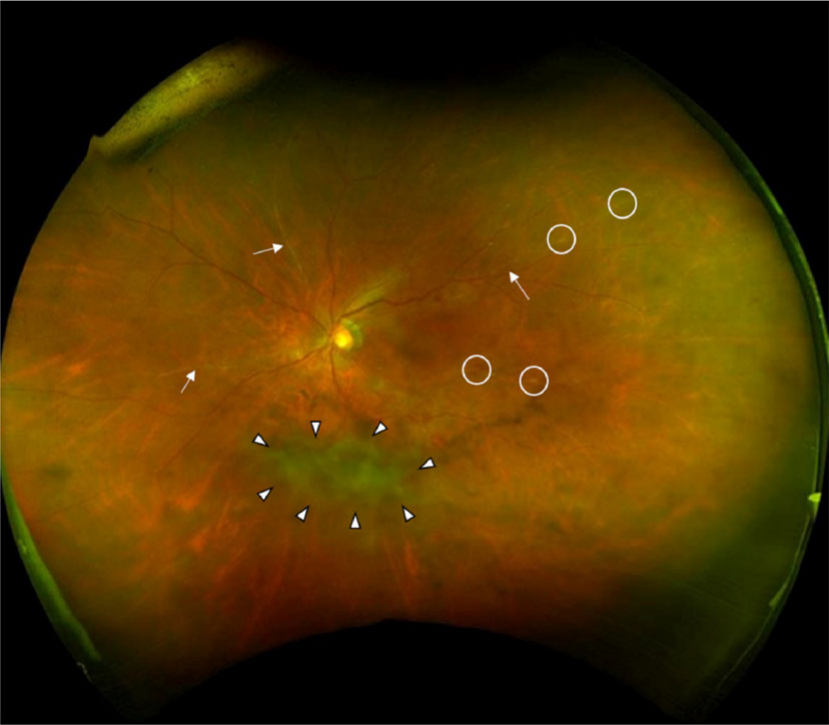
A colour fundus photograph of the left eye showing a dense vitreous opacity (surrounded by arrowheads), perivascular exudates (circles), and sheathing of retinal arteries (arrows).

Although he had normal visual acuity, inflammatory cells were observed in the anterior segment of both eyes. Fundoscopy showed no remarkable finding in the right eye, but sheathing of retinal vessels and vitreous opacification in the left eye were noted. Accordingly, he was diagnosed as having intraocular inflammation (uveitis) with retinal vasculitis, an irAE related to pembrolizumab use (Fig. 2).

He received treatment with 1 mg per body weight, 70 mg oral prednisolone daily for one week, and rapidly, the symptoms were ameliorated. The dose of prednisolone was tapered to 30 mg and continued for only one week.

Subsequently, the treatment was switched to topical corticosteroids (betamethasone eye drop), and triamcinolone acetonide (20 mg/0.5 mL) was injected periocularly as needed. There was no remarkable recurrence of intraocular inflammation throughout the follow‐up period. After oral prednisolone withdrawal, he had no symptom related to adrenal insufficiency while he had used small amount of topical corticosteroids.

A single pembrolizumab administration achieved a partial response (PR). After six cycles of the treatment, weight loss and a foul odour occurred, so treatment with pembrolizumab was paused. Six months has passed from discontinuing treatment with oral prednisolone. A blood test showed low level of adrenocorticotropic hormone (ACTH) (Table 1).

**Table 1:** Laboratory data (endocrinology).

After six cycles of treatment with pembrolizumab		After hormone therapy	
ACTH	<1 pg/mL (7.7–63.1)	ACTH	1.1 pg/mL (7.7–63.1)
Cortisol	0.9 μg/dL	Cortisol	1.1 μg/dL
TSH	3.66 μIU/mL (0.35–4.94)	TSH	20.15 μIU/mL (0.35–4.94)
FT4	0.86 ng/dL (0.7–1.48)	FT4	0.55 ng/dL (0.7–1.48)
		GH	0.15 ng/mL (0–3)
		IGF‐I	145 ng/mL (61–202)
		IGF‐I *Z*‐score	0.62
		Prolactin	9.8 ng/mL (3.58–12.78)
		LH	11.9 mIU/mL (0.79–5.72)
		FSH	24.8 mIU/mL (2–8.3)
		Testosterone	3.6 ng/mL (2.8–9.5)
		DHEA‐S	278 ng/mL (50–2530)

ACTH, adrenocorticotropic hormone; DHEA‐S, dehydroepiandrosterone‐S; FSH, follicle‐stimulating hormone; FT4, free thyroxine; GH, growth hormone; IGF‐I, insulin‐like growth factor 1; LH, luteinizing hormone; TSH, thyroid stimulating hormone.

Although extrinsic adrenal insufficiency was not excluded, ICI‐related hypophysitis and thyroiditis were primarily suspected in his clinical course. According to the judgement of our endocrinologist, six months had passed since the administration of prednisolone 30 mg was discontinued, and despite of no change in his medication other than pembrolizumab treatment, the sudden appearance of the adrenal insufficiency symptoms is leading to a diagnosis of ICI‐related hypophysitis. After hydrocortisone and levothyroxine replacement therapy, his symptoms were completely improved, indicating he has adrenal insufficiency and hypothyroidism. There was no change in the low level of ACTH after hormone therapy (Table 1).

Although discontinuation of pembrolizumab treatment was terminated at the request of the patient, there was no evidence of the relapse of lung cancer after 15 months (Fig. 1C, D). We report this case in which a variety of adverse events occurred, which were managed through smooth cooperation with other departments, and the lung cancer has not recurred.

## Discussion

Retinal vasculitis is included in posterior uveitis, and is rare compared to anterior uveitis as an irAE [1]. In some cases, treatment with oral steroids is preferred, and in other cases, only topical steroid treatment is performed. In one report, steroid treatment at the time of the first ICI administration may be disadvantageous because of the poor efficacy of ICI [2]. Another report showed that in patients with hypophysitis due to ipilimumab, cytotoxic T‐lymphocyte‐associated protein 4 (CTLA‐4) antibody, the therapeutic effect of ipilimumab was inferior in the group that used 7.5 mg or more of prednisolone compared to that in the group that used 7.5 mg or less, so high‐dose steroid treatment may not be beneficial in patients treated with ICIs [3]. Based on this information, although high‐dose steroid treatment was necessary in this case, we attempted to switch from systemic administration of steroids to topical treatment as early as possible and succeeded.

The patient in this case developed hypophysitis, thyroiditis, and retinal vasculitis. As far as we have observed, no cases of multiple irAEs involving ocular lesions and endocrine disorders have been reported. We reported that an improved long‐term prognosis may be expected in cases with PR at the onset of an irAE, and a therapeutic effect may be obtained without re‐administration in those cases [4]. In addition, two or more irAEs were reported to significantly prolong the progression free survival (PFS) and overall survival (OS) of patients treated with ICIs compared to one irAE episode [5]. In this case, discontinuation of treatment with pembrolizumab did not lead to the relapse of lung cancer.

There are various treatments depending on the patient, and it is necessary to consult specialists and cooperate with them for the correct treatment. Although our patients had multiple irAEs, he was able to be treated promptly through smooth cooperation with other departments.

In this case, three irAEs occurred, and there is a possibility that the survival period can be expected to be extended because the patient has not experienced recurrence at present. Further research is needed on irAEs and the effects of ICIs.

### Disclosure Statement

Appropriate written informed consent was obtained for publication of this case report and accompanying images.

### Author Contribution Statement

Motoko Tachihara was involved in design of the work and interpretation of data. All authors revised the manuscript, approved the manuscript to be published, and agreed to be accountable for all aspects of the work in ensuring that questions related to the accuracy or integrity of any part of the work are appropriately investigated and resolved.
